# The Limits of Dental Therapeutics

**Published:** 1901-12-15

**Authors:** E. L. Clifford

**Affiliations:** Chicago


### THE LIMITS OF DENTAL THERAPEUTICS.

BY DR. E. L. CLIFFORD, OF CHICAGO.

Read at the Joint Meeting of Northern Indiana and Southwestern Michigan Dental Societies, September 25th, 1901.

The mariner must know the currents of the sea he sails, and watch the winds that press upon his ship, or the vessel may be driven through the mists upon some dangerous shore, while he, absorbed in daily routine, reckless pleasure or unthinking toil, is quite unconscious of the peril which proper care and watchfulness might have averted. So we, who sail life's sea, must know the movements of our time to save ourselves from a state of wreck and guide the ship toward her port through smooth, safe waters, unshaken by the tempest.

Some of the currents in the sea of life to-day are so powerful and so obvious, that even the most careless voyager can scarcely fail to note them, though he may not understand them.

To imagine the difference of conditions now and in the early years of the past century, only a sudden and actual transfer, in full maturity of faculty, from one life to the other, could give a really vivid sense of the change. A Stevenson, a Howells, a Doyle, a Twain or a Bellamy might do justice to the theme, but no mild scientific or historical sketch can give more than a faint conception of the marvelous transformation wrought by the intellectual and material development of the nineteenth century.

Perhaps the nearest approach to a realization of the change above referred to, without the aid of the novelist, might be obtained by devoting a month or two to picturing in our thought the consequences of sweeping into oblivion all the inventions and discoveries—scientific, educational, moral, political, industrial and social achievement of the past century.

Think of life without a railroad—only a stage coach to carry our letters and ourselves; of a city without a street car or a bicycle, a cooking stove or a furnace, a gas jet or electric light, or even a kerosene lamp; a land without photographs or Christmas cards; of striking a flint and steel whenever you wanted a light. Science, industry and invention have changed the times

—have harnessed steam and electricity to do the world's work and develop hundreds of utilities and comforts to deepen and broaden modern life. George Washington never rode in a trolley car, or even a horse car, or talked over a telephone; never saw a locomotive or a steamboat, an elevator or an arc light, a department store, a pile driver, an asphalt road, a phonograph, a moving picture, or even a photograph. Ben Franklin, one of the wisest men of the olden time, wrote his letters with a quill and blew on the ink to dry it. He did not know enough to use a blotter, and never dreamed of a fountain pen or a typewriter. He never smelled chloroform or ether, or had a rebellious tooth pulled without pain by the aid of nitrous oxide gas or cocain. Napoleon could not send a telegram or ship his troops by rail, could not secure breech-loaders or smokeless powder or submarine ships, or take kodak views of his battles, or quell an insurrection in his appendix by having it cut out without danger from nervous shock or from blood poisoning. Hundreds of thousands of lives could have been saved in Napoleon's wars if the use of anesthetics and antiseptics had been known.

Knowledge has marvelously increased and has been diffused among all classes to a degree unknown in former times. Almost all sciences are developments of recent years; in fact, the whole body of modern science, except pure mathematics and the rudiments of astronomy, physics and chemistry, is the creation of the nineteenth century. The spectroscope has analyzed the stars; the Roentgen rays have lighted up the innermost recesses of the human body and made it easy to see through even the least transparent man. The camera has registered more than the eye can see, the phonograph more than the ear can hear. With the telegraph, telephone, and phonograph, color photography, the vitascope and kinetophone, one can bring the world to our doors. Electrical and magnetic developments, the new surgery and the germ theory of disease, the results of hypnotism and psychic research, the localization of the functions in the brain, the scientific interpretation of history, embryology and organic evolution, have given us a totally new conception of the universe and man. To hold the records of phenomena that science has studied, and the laws that have been deciphered, requires a library a thousand times as big as the library of science a hundred years ago. A high-school boy to-day knows more about the laws of nature, life, mind, history and society, than the greatest savant of the eighteenth century. A hundred years ago ignorance held the chief domain. The area in the light was but a speck to the long stretches in the dark.

The above thoughts, culled from a recent literary magazine, strike me as being so thoroughly in accord with the professional status of to-day that I have borrowed them as an introduction to the views I would place before you. As an advocate of more extended therapeutical knowledge and practice, I recognize fully the disputed foundation upon which I would build, the opposition with which I shall meet; but an article upon the subject in a recent professional journal, and the report of the discussion thereon, has prompted me to change the subject that I first selected for your consideration and, although a step backward, make an humble effort to add another stone, and so strengthen, if possible, the foundation upon which our future must stand.

In the early years of our specialty, practitioners, though in somewhat of a crude way sometimes, performed almost all of the operations and utilized almost all of the topical applications, though ignorant of our present theories, that the modern dentist of to-day applies.

Later knowledge has increased our success, has increased our methods and facilities, but has not enlarged our scope or field of practice. Judging from the discussion above referred to, our "limits of practice" stand to-day where they did forty years ago, and some fogies in the field would keep them there. Our abilities and our knowledge certainly have increased. Shall we always tread the beaten track?

What does the term "Therapeutics" mean? What does the word "limits" mean? What does a recognized college of dental surgery demand of its applicants for graduation? What rights and privileges does a diploma confer upon its holder? What rights and privileges are guaranteed to a person by the government under which he or she lives?

Therapeutics is defined as that science which treats of the curing of diseases. What is disease? Any deviation from the normal. "Limits" is defined in our most recent dictionaries as a boundary; a threshold; that which limits, bounds, or circumscribes; a border; the utmost extent; a restraint. In mathematics, a quantity toward which a varying quantity may approach but which it can not pass.

A recognized college of dental surgery to-day requires of its graduates not a limited, but a full and complete knowledge of anatomy, physiology, histology, embryology, pathology, general therapeutics and medical chemistry, together with a further and special knowledge of all these general sciences in their local and more special bearing and adaptation. Is the holder of the D.D.S. degree of 1901 a person expected to "fix and repair teeth,'' or would he be deemed culpable were he not able to recognize and direct treatment for any or all of the many and varying pathological lesions, the result of either local or general irritation, which manifest themselves within the oral cavity of his fellow man? Dr. Brophy, in his discussion on Dr. Peck's paper (see August *Review*, p. 827 *et seq.*), so ably answers this query of my paper that I refrain, and only refer the same to you. The rights and privileges conferred upon the holder of a diploma from a scientific institution, honestly obtained, are to use the knowledge he has gained in the pursuit of an honorable calling to gain a livelihood and properly maintain and support himself and those whom God has intrusted to his care.

If we answer the query in regard to what constitutes a ''dentist'' of to-day in the affirmative, for heaven's sake let us recognize the demands of the times at once, and split up, and hail the advent of the ''stomatologist'' or the ''orist.'' This I believe, appreciating the immensity and the ramification of knowledge and ability demanded of the modern dentist, must be the outcome and the result of the present discussion. A mechanic is not a therapist, nor need he be; and *vice versa*; but if a single person is to claim the field with its privileges he *must* prepare himself for the *demands* of the same. Talk is cheap, and there seems to have been much wasted in the discussion referred to.

Boiling it down, sifting and analyzing the precipitate, we find that two questions seem to predominate: First, shall the student be taught sufficient of the fundamentals of general medicine to know disease when he sees it?—and second, if he has been taught, has he the right, and is it his prerogative to use it? About the first of these questions there does not seem to be so much difference of opinion, and Dr. Peck has answered that portion in his paper forcibly, ably and *quantum sufficit*. The separation as to the position of practitioners seems to hover more about the second question. To answer this portion fully would require more time and patience than you have at hand, and far more skill with the pen than I am possessed of. I sift out a few thoughts which I drop for your deliberation, in short and precise statements, and trust to the discussion to clothe their nakedness and beautify their appearance.

I have always believed, I have always contended, that the dentistry of the past has been, and should have been, a specialty of the general practice of medicine; and I believe the dentistry of the present and future, when all of its possibilities are appreciated, *must* likewise be. I believe that the students must be educated with this fact thoroughly impressed upon them. I believe that there should be *two* divisions in the field now known as dentistry: prosthetics and therapeutics; and that they may be known in the future as "dentistry" and "stomatology." That the demands of the two divisions differ materially in their requirements, though both essentially related.

I said that "words had been wasted." There seems to be a disposition and a determination upon the part of the opponents of enlarging the dental sphere to harp upon the fact of "interference with our medical confreres," upon the possibility of the dental specialist overstepping the bounds and encroaching upon the field of "special" therapeutics. No writer or speaker in the literature of the past has ever claimed any such desire or tendency. It is not the *special* therapeutics of any particular disease that should command the attention of any specialist, save those of the *special*

conditions he is called upon to consult about or treat. But it is claimed, and very justly so, I believe, that where general pathology so affects or retards the re-establishment of the normal health status that general therapeutics is indicated, required or demanded for any special case, that any specialist, no matter what his field, should be competent to recognize the conditions existing, and that it is not only his right and privilege to exercise and use his knowledge, but his unbounded duty to do so. Dr. Brophy tells us that "the curriculum of our dental schools has broadened, and is yet broadening and including other branches of learning." What for? Shall the sphere of usefulness be contracted? We are told that the specialist has no right to treat rheumatism, gout, scrofula, tuberculosis, fecal impaction, etc. Who said he had? What specialist wants to treat these specific diseases? But if, in the course of Nature's laws, the same lowered vitality, and the same inability on the part of the patient rendering him unable to throw off toxins which are causing pathological lesions, which are brought to our attention for treatment and which manifest similar symptoms to those producing the diseases above mentioned, and are recognized as etiological factors in our special sphere—we do claim that it is the duty of the dental specialist to recognize these factors, appreciate their importance, and to remove them if possible. It is for a knowledge of *general* pathology that we would plead, and for the practice of *general* and *natural* therapeutics which we would claim as our right, our privilege and our duty. Experience teaches us that a weakened condition or a perverted function of any of the great emunctories of the body show signs and symptoms and cause sympathetic and concomitant manifestations in the tissues of the oral cavity
t as well as elsewhere. This is not the fault of the specialist; it might be considered the misfortune of the general practitioner or the patient. A little tact on the part of the specialist or the consultant, however, would go far to avoid any clash and smooth the road to intelligent relief. The general practitioner usually seeks the assistance of the specialist because he has felt his weakness or because he does not wish to further handle the case; and to refer a new patient to a strange physician for diseases of the mouth, except in special cases, which would be the exception rather than the rule, has in my experience been anything but satisfactory.

The field of the stomatologist should therefore be limited only by his knowledge, his capacity, or his desire; recognizing at all times the rights and privileges of others, and keeping in constant view the very best interests of the patient whose confidence and desire for relief has caused him to ring our office bell.

Reverting to the introduction of this paper, pause for a moment, and imagine if you can a dental office of to-day, in the light of our present knowledge, without steam, gas and electricity, without dental engine, automatic mallets, rubber dam, cohesive and sponge gold, fountain cuspidors, saliva ejectors, etc.; without general and local cardiac, nerve and respiratory stimulants, depressants, antiseptics and disinfectants, hæmostatics, diluents, antiphlogistics, diuretics and cathartics. Call up the spirit of your honored father who wrapped the cloak of immortality about his aching back some thirty years ago, who had rested from his labors while the ingenuity, perseverance and science of his son has kept apace with constant progress; let him step suddenly into your private office, view your surroundings, listen to your theories and advancement, and then stand aghast, if you will, at his consternation when you say to him: "With all these, with all my knowledge, the definition in our lexicons of to-day for the word dentist is 'a man who fixes and repairs teeth.'"

Shades of our ancesters, sleep on. May the spirits of successors, broadening to a fuller appreciation of our capabilities, hover around and encourage more and more the ambitions of future aspirants, until the world showers justice at last upon the shoulders of the worthy.

> "Grief dallied with no law nor limits knows."
>
> —*Waller.*

> "We wish removed what standeth in our light
> And nature blame for limiting our sight."
>
> —*Shakespere.*

